# Supplementary material for: Implementation of a care manager organisation and its association with antidepressant medication patterns: a register-based study of primary care centres in Sweden
Source: BMJ Open. 2021 Mar 5;11(3):e044959. doi: 10.1136/bmjopen-2020-044959 (PMC7938985; doi:10.1136/bmjopen-2020-044959)
Supplement: Author's manuscript [file bmjopen-2020-044959.draft_revisions.pdf]

BMJ Open is committed to open peer review. As part of this commitment we make the peer review history of every article we publish publicly available.

When an article is published we post the peer reviewers' comments and the authors' responses online. We also post the versions of the paper that were used during peer review. These are the versions that the peer review comments apply to.

The versions of the paper that follow are the versions that were submitted during the peer review process. They are not the versions of record or the final published versions. They should not be cited or distributed as the published version of this manuscript.

BMJ Open is an open access journal and the full, final, typeset and author-corrected version of record of the manuscript is available on our site with no access controls, subscription charges or pay-per-view fees (http://bmjopen.bmj.com).

If you have any questions on BMJ Open's open peer review process please email info.bmjopen@bmj.com

BMJ Open

Antidepressant medication use after implementation of a care manager organisation – a register-based study of primary care centres in Sweden

Journal:	*BMJ Open*
Manuscript ID	bmjopen-2020-044959
Article Type:	Original research
Date Submitted by the Author:	17-Sep-2020
Complete List of Authors:	Sandheimer, Christine; University of Gothenburg Sahlgrenska Academy, Social medicine, School of Public Health and Community Medicine<br>Björkelund, Cecilia; University of Gothenburg Sahlgrenska Academy, Primary Healthcare, School of Public Health and Community Medicine; Research and Development, Primary Healthcare, Region Västra Götaland<br>Hensing, Gunnel; University of Gothenburg Sahlgrenska Academy, Social medicine, School of Public Health and Community Medicine<br>Mehlig, Kirsten; University of Gothenburg Sahlgrenska Academy, Lifecourse Epidemiology, School of Public Health and Community Medicine, Institute of Medicine<br>Hedenrud, Tove; University of Gothenburg Sahlgrenska Academy, Medicine use & Pharmaceutical policy, School of Public Health and Community Medicine, Institute of Medicine
Keywords:	PRIMARY CARE, Quality in health care < HEALTH SERVICES ADMINISTRATION & MANAGEMENT, PSYCHIATRY, CLINICAL PHARMACOLOGY

SCHOLARONE™
Manuscripts

I, the Submitting Author has the right to grant and does grant on behalf of all authors of the Work (as defined in the below author licence), an exclusive licence and/or a non-exclusive licence for contributions from authors who are: i) UK Crown employees; ii) where BMJ has agreed a CC-BY licence shall apply, and/or iii) in accordance with the terms applicable for US Federal Government officers or employees acting as part of their official duties; on a worldwide, perpetual, irrevocable, royalty-free basis to BMJ Publishing Group Ltd ("BMJ") its licensees and where the relevant Journal is co-owned by BMJ to the co-owners of the Journal, to publish the Work in this journal and any other BMJ products and to exploit all rights, as set out in our licence.

The Submitting Author accepts and understands that any supply made under these terms is made by BMJ to the Submitting Author unless you are acting as an employee on behalf of your employer or a postgraduate student of an affiliated institution which is paying any applicable article publishing charge ("APC") for Open Access articles. Where the Submitting Author wishes to make the Work available on an Open Access basis (and intends to pay the relevant APC), the terms of reuse of such Open Access shall be governed by a Creative Commons licence – details of these licences and which Creative Commons licence will apply to this Work are set out in our licence referred to above.

Other than as permitted in any relevant BMJ Author's Self Archiving Policies, I confirm this Work has not been accepted for publication elsewhere, is not being considered for publication elsewhere and does not duplicate material already published. I confirm all authors consent to publication of this Work and authorise the granting of this licence.

For peer review only

Antidepressant medication use after implementation of a care manager organisation – a register-based study of primary care centres in Sweden

Christine Sandheimer[1*], Cecilia Björkelund[1,2], Gunnel Hensing[1], Kirsten Mehlig[1], Tove Hedenrud[1]

Affiliations

[*]Correspondence: Christine.sandheimer@gu.se

[1]Social medicine, School of Public Health and Community Medicine, Institute of Medicine, The Sahlgenska Academy, University of Gothenburg, Gothenburg, Sweden

[1]Primary Healthcare, School of Public Health and Community Medicine, Institute of Medicine, The Sahlgenska Academy, University of Gothenburg, Gothenburg, Sweden

[1]Lifecourse Epidemiology, School of Public Health and Community Medicine, Institute of Medicine, The Sahlgenska Academy, University of Gothenburg, Gothenburg, Sweden

[1]Medicine use & Pharmaceutical policy, School of Public Health and Community Medicine, Institute of Medicine, The Sahlgenska Academy, University of Gothenburg, Gothenburg, Sweden

[2]Research and Development, Primary Healthcare, Region Västra Götaland, Sweden

Word count: 3651

For peer review only - http://bmjopen.bmj.com/site/about/guidelines.xhtml

Abstract

Objective To evaluate the implementation of a care manager organisation (CMO) for common mental disorders (CMD) and its effects on antidepressant medication use on primary care centre (PCC) level, compared to PCCs without a CMO (i.e. care as usual ´CAU´). Moreover, to determine whether establishing a CMO at the PCC is associated with antidepressant medication use that is more in accordance with guidelines.

Design Register-based study on PCC level.

Setting Primary care in Region Västra Götaland (VGR) in south-west Sweden.

Participants All PCCs in the region. PCCs were analysed in three subgroups: PCCs with a CMO (CMO, n =68), PCCs without a CMO (CAU, n = 92) and PCCs that shifted from CAU to CMO during 2016 (Shift, n = 42).

Outcome measures Proportion of inadequate medication use (PIMU), defined as 0-179 Daily Defined Doses, among patients 18 years and older with a CMD diagnosis who received care at a PCC in VGR during 2015 and 2016. Register data on dispensed antidepressant medication per PCC was obtained from the regional prescribed drug database Digitalis. PIMU was analysed through generalised linear regression and a linear mixed-effects model.

Results In both years, PCCs with CMO had about 6% lower PIMU compared to PCCs with CAU (-6.1%, ns versus -6.4%, p = .02). These differences were explained by higher PIMU in privately run PCCs. PCCs with Shift, dominated by private PCCs, had a significant decrease in PIMU over time (p = .01) compared to the other PCCs.

Conclusions In general, PCCs in VGR followed the guidelines to a high extent both with and without the introduction of a CMO. However, public PCCs were more consistent in antidepressant use compared to private PCCs that gained more by introducing a CMO. Future studies should investigate the long-term effects of a CMO in Swedish primary care.

For peer review only - http://bmjopen.bmj.com/site/about/guidelines.xhtml

Keywords

Care manager, collaborative care, common mental disorders ‘CMD’, primary care, care as usual ‘CAU’, antidepressant use

Strengths and limitations of this study

- This is the first study aimed at assessing the implementation of a care manager organisation in Swedish ordinary primary care (PC)
- Register data on antidepressant medication use per primary care centre (PCC) is a strong quality indicator in the assessment of the PCC’s adherence to clinical guidelines
- The use of register data made it possible to include all PCCs in the south-west of Sweden
- As primary healthcare is context bound, the generalisability of the findings from this study may be limited
- As the differences between the PCCs and over time were small, future studies should include a longer time period to be able to distinguish the long-term effects of a care manager organisation in Swedish PC

For peer review only

Introduction

Common mental disorders (CMDs) such as depression, anxiety syndromes, and adjustment and stress-related disorders, are major causes of the disease burden world-wide [1]. In high income countries, such as most European countries and the United States (US), approximately 18 percent of the population experience a CMD during a year [2]. The economic burden of CMDs contain both direct costs, in the forms of healthcare visits and medications, and indirect costs, such as sickness absence and production loss [3].

Regarding healthcare visits, individuals with CMD often seek care at the primary care (PC) as the first contact with the healthcare system and it is in the PC the majority are treated [4–6]. In spite of this, for several years research has shown that the PC systematically fails to identify patients with CMD [4], especially when the patient seek care for a somatic complaint [7]. Moreover, the treatments for CMDs, the most common being psychotherapy and antidepressant medication use, have been shown to be inadequate and to not always follow established clinical treatment guidelines [8]. A suboptimal use of antidepressant medication increases the risk of relapse and might adversely affect the treatment outcome [9]. Research has also shown that just starting patients on antidepressant medications without the support and continuity given in coordinated and collaborative care only helped a minority of patients with depression [10].

During the last two decades, several countries have started to implement collaborative care models (CCM) in their PC to improve the care given to patients with CMD [11,12]. The CCM is a system level intervention often including four key components: i) an interdisciplinary approach, ii) structured care planning, iii) a systematic follow-up of the patient, and iv) increased communication between healthcare personnel [13]. The model was adapted from the Chronic Care Model of disease management developed by Wagner in 1996 [14]. Wagner and colleagues suggested that team-based care, incorporating the four above-mentioned

For peer review only

components and where a non-physician caregiver (e.g. a nurse) had the main responsibility for the patient, would make the care more efficient and would improve treatment outcomes [14].

In 2012, the Swedish Council on Health Technology Assessment [15] stressed a need for studies in Swedish PC to determine whether the CCM with special trained care managers, hereafter called the care manager organisation (CMO), could result in the same positive effects as shown in other PC settings [16,17]. Thus, a first pragmatic, cluster-randomised controlled trial (PRIM-CARE) with 23 primary care centres (PCC) in Region Västra Götaland in the south-west of Sweden was conducted in 2014 [18]. The results from the PRIM-CARE study showed positive effects of the CMO regarding symptom remission and a more consistent antidepressant medication use compared to care as usual (CAU) [18]. After the intervention study a new phase was initiated with the aim of implementing a CMO more widely in the region.

Even though the positive effects on depression and anxiety outcomes of collaborative care in the PC have been well established by now, the evidence base regarding quality assessment of important components in the CCM is poor [19]. One important process variable for a quality assessment is to measure the continuation of antidepressant medication use [20]. A common variable to measure continuity of medication is Defined Daily Doses (DDD) defined as: *"The assumed averaged maintenance dose per day for a drug used for its main indication in adults."*[21]. According to clinical guidelines, antidepressant medication use for depression and anxiety disorders should continue for at least six months (180 days) to minimise the risk of relapse of symptoms and unnecessary prolongation of the disorder [22,23].

The present study is the first in Sweden that aimed to quality assess the CMO in Swedish PC on a system level. Our aim was to investigate the association of a CMO with antidepressant medication use, as a quality indicator, at PCC level. We wanted to investigate the

For peer review only

antidepressant use in PCCs with and without a CMO both in 2015 (before the implementation) and in 2016 (when the implementation was accomplished), as well as the longitudinal changes in the antidepressant use among the PCCs from 2015 to 2016. Moreover, we also wanted to assess whether the implementation of a CMO for patients with CMD at the PCC, compared to CAU (PCCs without a CMO) would be associated with antidepressant medication use that was more in accordance with established national clinical guidelines.

Methods

Study design and context

The present study was based on aggregated register data at PCC level and was a collaboration between the research programme "New Ways – mental health at work" and the research project PRIM-CARE that is part of the research platform "Ways-of-life, stress and mental health in the primary care context" at the School of Public Health and Community Medicine, University of Gothenburg, Sweden and Research and Development Primary Healthcare, Region Västra Götaland.

Study setting and participating PCCs

This study was conducted in the south-western part of Sweden, in the Västra Götaland Region (VGR) with a population of 1.7 million (17 % of the Swedish population).

VGR is not just a geographical area in Sweden but also an organisational entity, responsible for financing and producing the healthcare in the region. The number of PCCs in VGR has varied over the years but is around 200, with a small majority having public management.

Care manager

For peer review only

For peer review only - http://bmjopen.bmj.com/site/about/guidelines.xhtml

The care manager is a designated nurse that has completed special training in care management for patients with CMD, especially mild to moderate depression.

The implementation process in VGR started in the fall of 2015, with the first nurses finishing their examination in January 2016.

The work process of the care manager is as follows: when the patient is diagnosed with CMD by the primary care physician, collaborative care in addition to usual care is offered to him/her through the use of a care manager during an initial visit to draw up a joint care plan. The care plan is followed-up by regular telephone contacts. During the weekly contacts with the patient, that continue for around three months, the care manager assesses the patient's CMD symptoms, and supports and engages the patients in following their treatment, in taking their medications according to the prescription, supervising potential side effects of medications and deciding if there is a need to change the dosage or type of medicine.

The CMO involves the whole staff at the PCC. All personnel are given brief training on the treatment guidelines for CMD and how to collaborate with the care manager nurse. The CMO aims at improving the collaboration among personnel, and at providing enhanced patient information and patient education about treatment and treatment options. More details about the care manager function and organisation can be found in a previously published article by Björkelund et al [18].

Care as usual

CAU for patients with CMD in Swedish PCCs can consist of visits to different healthcare professionals (such as physicians, nurses, psychologists/psychotherapists, or physiotherapists). The treatment should follow the guidelines set by the region which are based on the national evidence-based clinical guidelines for CMD [24]. These guidelines include face-to-face psychotherapy, antidepressant medication treatment and/or sick listing.

For peer review only - http://bmjopen.bmj.com/site/about/guidelines.xhtml

Outcome measure

The outcome measure for this study was the number of patients aged 18 and older diagnosed with F32, F33, F40, F41 or F43, (i.e. mild - moderately severe depression, anxiety syndrome, social phobia and/or stress induced mental ill health) that dispensed an antidepressant medication for between 0 to 179 days, i.e. less than six months treatment, as obtained from each PCC. 0-179 DDD was defined as inadequate medication use in accordance with clinical treatment guidelines for CMD [22,23]. As the outcome variable we used the measure *Proportion of inadequate medication use* (PIMU). Antidepressants (Anatomical Therapeutic Chemical 'ATC' code N06A) was the medication group of interest in this study as these medications are used in the treatment of both depression and different anxiety syndromes [25].

Data and study period

We collected register data on healthcare use and dispensed medications among patients aged 18 and older with a CMD diagnosis who were receiving care at a PCC in the region within the study period. The data was collected on an aggregated level with information per PCCs and not per patient. The data was obtained from the healthcare database VEGA and the regional prescribed drug register Digitalis. Both are managed by VGR. VEGA contains health care information about the inhabitants in the VGR; all health care consumption is included even the care that has been consumed outside the region. Digitalis contains both information on dispensed medications prescribed in the region from one of the region's PCCs but dispensed in any Swedish pharmacy as well as information about prescriptions dispensed by the region's inhabitants, even those that are prescribed outside the region.

For peer review only

For peer review only - http://bmjopen.bmj.com/site/about/guidelines.xhtml

As analytic sample, we included PCCs active during both 2015 and 2016 to have comparable groups in the statistical analyses. This led to the exclusion of 22 PCCs that had either started up or shut down their health care activity during 2016. The PCCs were divided into three groups for analysis based on their CMO status: *CMO* (PCCs that implemented the CMO in January 2016 and continued having a CMO during all of 2016), *Shift* (PCCs that implemented the CMO at some point during 2016), and *CAU* (PCCs without a CMO in either 2015 or 2016). Each PCC group was controlled for three different covariates: i) if they had a private or public management, ii) number of listed patients, and iii) proportion of patients with a CMD diagnosis (number of patients with a CMD diagnosis divided by number of listed patients).

The study period was January to December 2015 (before implementation of a CMO) and January to December 2016 (when the implementation was accomplished).

Statistical analyses

The characteristics of the PCCs by CMO status in 2015 and in 2016 is presented in Table 1, and *p*-values of group differences were obtained with the non-parametric Kruskal Wallis test. As the distribution of the outcome variable PIMU was skewed to the right we log-transformed the variable which yielded normally distributed residuals in linear regression models. Cross-sectional analyses used linear regression of log PIMU on the categories of CMO status (ref = CAU), and further adjusted for private management (ref = public management). The resulting beta-coefficients β were further transformed to give the relative difference in percent for PIMU in PCCs with CMO compared to CAU, calculated as (exp(β) – 1) x 100%. Cross-sectional analyses were performed for both 2015 and 2016.

To investigate the effect of CMO status on the longitudinal change in PIMU within PCC groups we implemented a linear mixed-effects model. The model took into account the

For peer review only

correlation between repeated measures for the same PCC [26]. The result is illustrated in a figure (Fig. 1) with *p*-values of differences within PCC groups. The model for longitudinal change and CMO status was adjusted for CMO status, year, the interaction between CMO status and year, and further adjusted for private management status and the proportion of patients with CMD diagnosis.

Analyses were performed using IBM SPSS Statistics® version 25 and 26. Statistical significance was set at 0.05 (two-sided tests) with presentation of 95 % confidence intervals.

Patient and Public Involvement

Neither patients nor the public were involved in the design or conception of this study.

Results

Table 1 presents the description of PCCs by CMO status from baseline (2015) and after accomplished implementation (2016). The descriptive data in Table 1 include number of visits of the study population to physicians and nurses as these measures are correlated with the introduction of a CMO. A total of 190 PCCs were included in the study, of which 81 were classified as CAU, 42 as Shift, and 67 as CMO.

Public or private management was highly correlated with CMO status (p = <0.001, data not shown); only sixteen private PCCs (24%) had implemented a CMO at the start of 2016 compared to 51 (76%) public PCCs. Among the PCCs without a CMO (CAU), 46 % were public and 54 % private. Shifts were mostly private (62 %, n = 26) compared to public (38 %, n = 16). The proportions of patients with a CMD diagnosis (of total number of listed patients) per PCC were about 9 % (2015) to 10 % (2016). Visits to nurses and physicians by the study population were higher in 2016 compared to 2015 in all three CMO status groups, but only visits to nurses had a statistically significant increase over time across the three categories of

For peer review only - http://bmjopen.bmj.com/site/about/guidelines.xhtml

CMO status (p = <0.01, data not shown). All PCC groups had lower values of PIMU in 2016 compared to 2015.

Table 1. Description of PCCs by CMO status (CAU[a] n = 81, Shift[b] n = 42 or CMO[c] n = 67) the year before implementation (2015) and the year when the implementation was accomplished (2016).

	Before implementation (2015)				Accomplished implementation (2016)			
	CAU[a] n (%)	Shift[b] n (%)	CMO[c] n (%)	*p*	CAU n (%)	Shift n (%)	CMO n (%)	*p*
PCCs with no. of listed patients								
<5000	18 (22)	7 (17)	13 (20)		18 (22)	7 (17)	12 (18)	
5000 - <7000	14 (18)	9 (21)	7 (10)		12 (15)	8 (19)	7 (10)	
7000 - <10.000	26 (32)	17 (41)	21 (31)		26 (32)	18 (43)	23 (35)	
≥ 10.000	23 (28)	9 (21)	26 (39)		25 (31)	9 (21)	25 (37)	
Mean value (SD)	8206 (3631)	8268 (3786)	8855 (3842)	*.53*	8452 (3594)	8442 (3749)	8921 (3802)	*.69*
PCCs with no. of patients with CMD diagnosis								
<500	23 (28)	10 (24)	15 (22)		16 (19)	10 (24)	13 (20)	
500 - <700	18 (23)	13 (31)	14 (21)		20 (25)	6 (14)	9 (13)	
700 - <1000	17 (21)	10 (24)	20 (30)		20 (25)	14 (33)	20 (30)	
≥ 1000	23 (28)	9 (21)	18 (27)		25 (31)	12 (29)	25 (37)	
Mean value (SD)	756 (344)	774 (411)	818 (394)	*.95*	820 (366)	858 (429)	901 (429)	*.25*
PCCs with no. of patients on antidepressants							67	
<300	23 (28)	11 (26)	13 (20)		17 (21)	11 (26)	11 (17)	
300 - <500	24 (30)	18 (43)	23 (34)		30 (37)	14 (34)	21 (31)	
500 - <700	21 (26)	6 (14)	20 (30)		18 (22)	9 (21)	18 (27)	
≥ 700	13 (16)	7 (17)	11 (16)		16 (20)	8 (19)	17 (25)	
Mean value (SD)	450 (223)	461 (241)	485 (233)	*.70*	487 (231)	499 (249)	528 (244)	*.57*
PCCs with no. of visits by patients with CMD to:								
Physicians[e], n[d]	80		66					
<1000	19 (24)	12 (29)	14 (21)		17 (21)	9 (21)	9 (13)	
1000 - <1500	22 (27)	12 (29)	15 (23)		14 (17)	11 (26)	15 (23)	
1500 - <2000	15 (19)	8 (19)	13 (20)		17 (21)	5 (12)	11 (16)	
≥ 2000	24 (30)	10 (23)	24 (36)		33 (41)	17 (41)	32 (48)	
Mean value (SD)	1660 (855)	1584 (994)	1839 (954)	*.27*	1873 (946)	1985 (1245)	2202 (1218)	*.35*
Nurses[e], n[d]	76	36	64		77	37	67	
<50	34 (45)	15 (42)	23 (36)		18 (23)	9 (25)	11 (17)	
50 - <300	22 (29)	15 (42)	17 (26)		25 (33)	19 (51)	25 (37)	
300 - <500	6 (8)	3 (8)	12 (19)		8 (10)	2 (5)	6 (9)	
≥ 500	14 (18)	3 (8)	12 (19)		26 (34)	7 (19)	25 (37)	
Mean value (SD)	293 (453)	191 (322)	291 (450)	*.61*	455 (588)	282 (368)	571 (690)	*.05*
Proportion of inadequate medication use (PIMU)								
% (SD)	32.8 (9.7)	33.8 (10.4)	30.3 (5.1)	*.15*	32.5 (8.3)	31.6 (5.3)	29.9 (4.0)	*.18*

[a]Care as usual, [b]Shifted from CAU to a care manager organisation, [c]Care manager organisation
[d]Number of valid observations

For peer review only

For peer review only - http://bmjopen.bmj.com/site/about/guidelines.xhtml

Table 2. Association between the proportion of inadequate medication use (PIMU) and PCCs' CMO status (CAU[a], Shift[b] or CMO[c]).

		**2015** (Baseline) Relative difference[+] % (95% CI)		**2016** Relative difference[+] % (95% CI)	
	n[d]	Unadjusted model	Adjusted for private PCC	Unadjusted model	Adjusted for private PCC
CAU	81	Ref.	Ref.	Ref.	Ref.
Shift	42	2.7% (-5; 11)	1.6% (-5.6; 9.4)	-1.5% (-7.8; 2.5)	-2.5% (-8.3; 3.7)
CMO	67	-6.1% (-12.3; 0.4)	-2% (-8.3; 4.7)	-6.4% (-11.6; -0.9)*	-2.6% (-7.9; 2.9)

[a]Care as usual, [b]Shifted from CAU to a care manager organisation, [c]Care manager organisation
[+]Relative difference in percent = [exp(β)-1] x 100 %.
[d]Number of valid observations
*p-value <0.05

Cross-sectional results (Table 2)

In both years, cross-sectional analyses showed that PCCs with CMO had about 6 % lower PIMU compared to PCCs with CAU, but the difference was only significant in 2016 (p = 0.02).

After controlling for private or public management the differences by CMO status reduced. The covariates *Number of listed patients* and *Proportion of patients with CMD diagnosis* did not have any effect on the association between PIMU and CMO status (data not shown). The association between CMO status and PIMU did not differ between private or public PCCs (p-value for interaction > 0.4 for both time points).

Figure 1 here

Longitudinal results

We used a linear mixed-effects model to investigate changes in PIMU between the three PCC groups with different CMO status over time (Fig. 1). The results showed no overall interaction between year and CMO status (p = 0.17, data not shown). As illustrated in figure 1, all three PCC groups had a decrease in PIMU over time but this change was only significant in PCCs with Shift (p = 0.01). This trend was also observed after further

For peer review only - http://bmjopen.bmj.com/site/about/guidelines.xhtml

adjustment for private management ($p = 0.01$) and for the proportion of patients with a CMD diagnosis ($p = 0.02$).

Discussion

The present study is the first in Sweden that, on the PCC level, has assessed a new care function with an organisational change in the Swedish primary care. The aim was to investigate the association between the implementation of a care manager organisation (CMO) in Swedish PC and antidepressant medication use as a form of quality indicator of the work to improve the care given to patients with CMD.

The main finding of this study was that a significantly lower proportion of patients with inadequate antidepressant medication could be shown for the PCCs that had implemented a CMO during the whole of 2016 compared to PCCs without a CMO. This finding was to a large extent explained by PCCs with private management, which showed the greatest impact of implementing a CMO for patients with CMD. Furthermore, only PCCs that shifted from CAU in 2015 to a CMO at some point during 2016 had a significant decrease of inadequate medication use over time. This PCC group was dominated by privately driven PCCs. Among PCCs that started implementation in late 2015 and had a CMO during the whole of 2016, the change was barely visible as these PCCs had a lower PIMU even before the implementation. Another finding was that publicly driven PCCs with and without a CMO did not differ in PIMU.

Approximately 70 % of the PCCs in all three groups had an antidepressant medication use that was in accordance with the recommendations in established clinical guidelines, i.e. medication treatment lasting six months or longer [22,23]. A point important to acknowledge is that not all individuals need a medication treatment that lasts more than 180 days. An ambition of 100 % medication use in accordance with treatment guidelines is therefore

For peer review only

unrealistic. Research has shown that about 15 % of Swedish patients do not collect their medications and the reasons behind are many [27]. The guidelines should be seen as recommendations not disregarding the patients' individual needs [27].

Nevertheless, the antidepressant use being in congruence with clinical guidelines and the absence of differences between the groups, could possibly be partially explained by the fact that PCCs in Region Västra Götaland (VGR) are bound by contract to deliver care according to regional clinical guidelines. The region has set up prescribing objectives for a rational medication use [28]. These objectives are found in a prescribing guidelines booklet available for all PCCs to help them develop good prescribing routines. This could be interpreted to mean that even a PCC without a CMO can deliver high pharmacological care quality that resonates well with the guidelines. Our assumption is supported by the findings from a study performed in the VGR in which primary care physicians' attitudes and use of the prescribing guidelines booklet were investigated [29]. The study found that the majority of physicians had positive attitudes towards the booklet and used it frequently. The same study also showed that physicians in private PCCs were more negative towards the booklet and did not adhere to the guidelines to the same extent as the physicians in the public organisations. This result could partly explain our finding that the public PCCs were stable in their medication use after the implementation of a CMO and mostly the private PCCs showed an improvement.

In the preceding intervention study (of the present study), the result showed that a care manager positively affected the antidepressant treatment in patients with depression [18]. The findings from the intervention study were in line with previous research from other healthcare contexts which has proved that support from a care manager during the treatment for CMD increases the frequency of adequate antidepressant medication use on the individual level [10,15,20,30].

For peer review only

For peer review only - http://bmjopen.bmj.com/site/about/guidelines.xhtml

Methodological considerations

The chosen design for this study possesses both strengths and potential limitations. A strength with a register based study is the reduced risk of bias due to low sampling, high attrition levels, recall bias and other methodological fallacies that are more frequent in survey studies [31]. The register databases used in this study; the health care database VEGA and the regional prescription drug register Digitalis, are comprehensive, systematic and updated on a regular basis. Digitalis is based on the same data as the national prescribed drug register in Sweden, enabling a national comparison of data. The use of data from VEGA and Digitalis allowed us to include all PCCs in the VGR of various sizes and management types, and from different rural and urban settings. There are approximately 1200 PCCs in Sweden, and this makes our sample, and thus our findings, generalisable on a national level. However, the applicability of our results to an international context could be limited since healthcare systems, and particularly, the primary care organisation, differ widely between countries [15]. A limitation with the use of aggregated data is the risk of information bias that is difficult to control for when we lack detailed information on the individual level [32]. The registers contain comprehensive information but they also require skills and experience of researchers to discern what the statistics embody and if the information is managed correctly. In our case, a thorough scrutiny of the data led to the exclusion of 22 PCCs that had extreme values of PIMU because the PCCs did not have a health care activity during the whole study period. As a result, we believe the reliability of our findings improved substantially.

Another methodological issue is the small observed differences between the PCC groups that could be due to the chosen period of study. The implementation process for the CMO started in January 2016 but the education for the care manager nurses began in late 2015. To have used 2015 as the baseline year and 2016 as the follow-up year could perhaps have led to the

For peer review only

For peer review only - http://bmjopen.bmj.com/site/about/guidelines.xhtml

absence of greater differences. A longer follow-up period of potential effects of the introduction of the CMO would have been preferable.

The last potential limitation in the present study is the absence of prescription data, i.e. information about what the physicians prescribed. A comparison between dispensed and prescribed medications to discern possible disparities in the data would have made a stronger quality indicator. Nevertheless, dispensed medication is a better indicator of medication use than prescribing data [33] as it gives us a clearer picture of the active behavior of the patient rather than the prescriber, and because many patients do not fill their prescriptions [27]. Hence, we believe that register data on dispensed medication was the better option for measurement as one of the tasks of the care manager is to help the patients to take their medicines according to the prescription guidelines.

Implications

Antidepressant medication treatment is influenced by several different factors that affect the outcome. These include both medical and patient-related factors such as accessibility, diagnostics and diagnostic process, guidelines, patient knowledge, and adherence [34,35]. Most of these factors can be influenced by the care manager [19] and thus an assessment of quality improvements achieved by a CMO should be evaluated not only on the individual patient level, but also on the PCC level by using healthcare registers. Assessments of complex interventions aiming at changing routine practice in primary care form an important knowledge base for decision-makers responsible for the provision and financing of healthcare. As this is the first study that assessed the implementation of a CMO in Swedish PC on PCC level with data when the organisational change was new, future studies are needed to further investigate the effects of the CMO on a long-term basis.

For peer review only

Conclusions

This quality assessment of the care manager organisation (CMO) for patients with CMD in Swedish primary care constitutes an important knowledge base in the further implementation process. The result of this study showed that, in general, PCCs in VGR followed the guidelines to a high extent both with and without the introduction of a CMO. However, public PCCs were more consistent in antidepressant use compared to PCCs with private management that gained more in introducing a CMO. However, future studies should include a longer time period to be able to distinguish the long-term effects of a CMO in Swedish PC.

Acknowledgements

The authors would like to thank Nashmil Arai for her excellent work on the data management.

Author Contributions

CB is the principal investigator of the project and was responsible for the design of the study. All authors took part in planning the analysis and the writing of the manuscript. CS had the main responsibility for conducting the analysis and the writing of the paper. KM supervised and assisted in the statistical analysis. All authors contributed to the interpretation of data and read and approved the final version of the manuscript.

Funding

This work was supported by the Swedish Research Council for Health, Working Life and Welfare (FORTE), grant number 2013-2216, and by grants from the Swedish state under the agreement between the Swedish government and the regions, the ALF-agreement (ALFGBG-722441) and Region Västra Götaland. The funding organ had no influence on the design of

For peer review only

the study, data collection, analysis or interpretation of data or writing of the report or the decision to publish the article. All authors were independent from the funder.

Competing interests

None declared.

Patient consent for publication

Not required.

Ethics approval

The collected register data used in this study was on an aggregated level with information per PCC and not per patient. No individuals could be identified and no informed consent was necessary. Ethical approval was received from the regional Ethical Review Board in Gothenburg, Sweden (Dnr: T566-17).

Provenance and peer review

Not commisioned; externally peer reviewed.

Data availability statement

Data are not publicly available due to Swedish law but are available from the authors upon reasonable request.

Open access

This is an open access article distributed in accordance with the Creative Commons Attribution Non Commercial (CC BY-NC 4.0) license, which permits others to distribute,

For peer review only

For peer review only - http://bmjopen.bmj.com/site/about/guidelines.xhtml

remix, adapt, build upon this work non-commercially, and license their derivative works on different terms, provided the original work is properly cited, appropriate credit is given, any changes made indicated, and the use is non-commercial. See: http://creativecommons.org/licenses/by- nc/4. 0/.

ORCID iDs

Christine Sandheimer http://orcid.org/0000-0002-2429-0669

Cecilia Björkelund http://orcid.org/0000-0003-4083-7342

Gunnel Hensing https://orcid.org/0000-0002-3457-2993

Kirsten Mehlig https://orcid.org/0000-0002-2653-0734

Tove Hedenrud https://orcid.org/0000-0003-2526-7097

References

1 WHO. Depression and Other Common Mental Disorders: Global Health Estimates. *World Heal Organ* 2017.

2 Steel Z, Marnane C, Iranpour C, *et al.* The global prevalence of common mental disorders: A systematic review and meta-analysis 1980-2013. *Int J Epidemiol* 2014;**43**:476–93. doi:10.1093/ije/dyu038

3 OECD. Health at a Glance: Europe 2018 (Summary in Spanish). 2018. doi:10.1787/fd41e65f-es

4 Sundquist J, Ohlsson H, Sundquist K, *et al.* Common adult psychiatric disorders in Swedish primary care where most mental health patients are treated. *BMC Psychiatry* 2017;**17**:1–9. doi:10.1186/s12888-017-1381-4

5 Lykkegaard J, Rosendal M, Brask K, *et al.* Prevalence of persons contacting general practice for psychological stress in Denmark. *Scand J Prim Health Care* 2018;**36**:272–

For peer review only - http://bmjopen.bmj.com/site/about/guidelines.xhtml

80. doi:10.1080/02813432.2018.1499494

6 Kessler RC, Demler O, Frank RG, *et al.* Prevalence and Treatment of Mental Disorders, 1990 to 2003. *N Engl J Med* 2005;**352**:2515–23.

7 Adamsson A, Bernhardsson S. Symptoms that may be stress-related and lead to exhaustion disorder: A retrospective medical chart review in Swedish primary care. *BMC Fam Pract* 2018;**19**:1–9. doi:10.1186/s12875-018-0858-7

8 Sinnema H, Terluin B, Volker D, *et al.* Factors contributing to the recognition of anxiety and depression in general practice. *BMC Fam Pract* 2018;**19**. doi:10.1186/s12875-018-0784-8

9 Donoghue J, Taylor DM. Suboptimal use of antidepressants in the treatment of depression. *CNS Drugs* 2000;**13**:365–83. doi:10.2165/00023210-200013050-00006

10 DeJesus RS, Angstman KB, Cha SS, *et al.* Antidepressant Medication Use Among Patients with Depression: Comparison between Usual Care and Collaborative Care Using Care Managers. *Clin Pract Epidemiol Ment Heal* 2013;**9**:84–7. doi:10.2174/1745017901309010084

11 Overbeck G, Kousgaard MB, Davidsen AS. The work and challenges of care managers in the implementation of collaborative care: A qualitative study. *J Psychiatr Ment Health Nurs* 2018;**25**:167–75. doi:10.1111/jpm.12449

12 Nieuwenhuijsen K, Faber B, Verbeek J, *et al.* Interventions to improve return to work in depressed people. *Cochrane Database Syst Rev* 2014;**2014**.

13 Gunn J, Diggens J, Hegarty K, *et al.* A systematic review of complex system interventions designed to increase recovery from depression in primary care. *BMC Health Serv Res* 2006;**6**:1–11. doi:10.1186/1472-6963-6-88

14 Wagner EH, Austin BT, Korff M Von, *et al.* Organizing Care for Patients with Chronic Illness. *Milbank Q* 1996;**74**:511–

For peer review only

For peer review only - http://bmjopen.bmj.com/site/about/guidelines.xhtml

44.https://www.jstor.org/stable/3350391?seq=1#metadata_info_tab_contents

15 Swedish Council on Health Technology Assessment (‘SBU’ Statens beredning för medicinsk utvärdering). Implementation support for psychiatric evidence in primary care. Report 211. Stockholm: 2012.

16 Rost K, Nutting P, Smith J, *et al.* Improving depression outcomes in community primary care practice: A randomized trial of the QuEST intervention. *J Gen Intern Med* 2001;**16**:143–9.

17 Gilbody S, Whitty P, Grimshaw J, *et al.* Educational and Organizational Interventions to Improve the Management of Depression in Primary Care: A Systematic Review. *JAMA* 2003;**289**:3145–51.

18 Björkelund C, Svenningsson I, Hange D, *et al.* Clinical effectiveness of care managers in collaborative care for patients with depression in Swedish primary health care: A pragmatic cluster randomized controlled trial. *BMC Fam Pract* 2018;**19**:1–10. doi:10.1186/s12875-018-0711-z

19 Moriarty AS, Coventry PA, Hudson JL, *et al.* The role of relapse prevention for depression in collaborative care: A systematic review. *J Affect Disord* 2020;**265**:618–44. doi:10.1016/j.jad.2019.11.105

20 Hudson JL, Bower P, Kontopantelis E, *et al.* Impact of telephone delivered case-management on the effectiveness of collaborative care for depression and anti-depressant use: A systematic review and meta-regression. *PLoS One* 2019;**14**:e0217948. doi:10.1371/journal.pone.0217948

21 WHO. Essential medicines and health products: The ATC/DDD Methodology. 2020.https://www.who.int/medicines/regulation/medicines-safety/toolkit_methodology/en/ (accessed 21 Feb 2020).

22 Cleare A, Pariante CM, Young AH, *et al.* Evidence-based guidelines for treating

For peer review only

depressive disorders with antidepressants: A revision of the 2008 British Association for Psychopharmacology guidelines. *J Psychopharmacol* 2015;**29**:459–525. doi:10.1177/0269881115581093

23 Swedish Medical Products Agency [Läkemedelsverket]. Pharmaceutical treatment of depression, anxiety syndrome and OCD amongst children and adults [Läkemedelsbehandling av depression , ångest- syndrom och tvångssyndrom hos barn och vuxna]. *Inf from Swedish Med Prod Agency [Information från Läkemedelsverket Swedish]* 2016;**6:2016**.

24 The Swedish National Board of Health and Welfare [Socialstyrelsen]. *National guidelines for care of depression and anxiety disorders [Vård vid depression och ångestsyndrom]*. Stockholm: : Socialstyrelsen 2017.

25 Swedish Medical Products Agency [Läkemedelsverket]. N06A Antidepressants. Pharm. B. [Läkemedelsboken. Swedish]. 2016.https://lakemedelsboken.se/kapitel/lakemedelsanvandning/trafik_riskfyllt_arbete_och_lakemedel.html?search=N06A&id=N06A-Antidepressiva-medel#N06A-Antidepressiva-medel (accessed 15 Jun 2020).

26 West BT. Analyzing longitudinal data with the linear mixed models procedure in SPSS. *Eval Heal Prof* 2009;**32**:207–28. doi:10.1177/0163278709338554

27 Freccero C, Sundquist K, Sundquist J, *et al.* Primary adherence to antidepressant prescriptions in primary health care: a population-based study in Sweden. *Scand J Prim Health Care* 2016;**34**:83–8. doi:10.3109/02813432.2015.1132884

28 Carlzon D, Gustafsson L, Eriksson AL, *et al.* Characteristics of primary health care units with focus on drug information from the pharmaceutical industry and adherence to prescribing objectives: A cross-sectional study. *BMC Clin Pharmacol* 2010;**10**:7–11. doi:10.1186/1472-6904-10-4

For peer review only

For peer review only - http://bmjopen.bmj.com/site/about/guidelines.xhtml

29 Axelsson MAB, Spetz M, Mellén A, *et al.* Use of and attitudes towards the prescribing guidelines booklet in primary health care doctors. *BMC Clin Pharmacol* 2008;**8**:1–8. doi:10.1186/1472-6904-8-8

30 Hudson TJ, Fortney JC, Pyne JM, *et al.* Reduction of patient-reported antidepressant side effects, by type of collaborative care. *Psychiatr Serv* 2015;**66**:272–8. doi:10.1176/appi.ps.201300570

31 Schwartz S. The fallacy of the ecological fallacy: the potential misuse of a concept and the consequences. *Am J Public Health* 1994;**84**:819–24. doi:10.2105/AJPH.84.5.819

32 Morgenstern H. Ecologic Studies in Epidemiology: Concepts, Principles, and Methods. *Annu Rev Public Health* 1995;**16**:61–81. doi:10.1146/annurev.publhealth.16.1.61

33 Wettermark B, Hammar N, MichaelFored C, *et al.* The new Swedish Prescribed Drug Register—Opportunities for pharmacoepidemiological research and experience from the first six months. *Pharmacoepidemiol Drug Saf* 2007;**16**:726–35. doi:10.1002/pds.1294

34 Kini V, Michael Ho P. Interventions to Improve Medication Adherence: A Review. *JAMA - J Am Med Assoc* 2018;**320**:2461–73. doi:10.1001/jama.2018.19271

35 Fischer MA, Stedman MR, Lii J, *et al.* Primary medication non-adherence: Analysis of 195,930 electronic prescriptions. *J Gen Intern Med* 2010;**25**:284–90. doi:10.1007/s11606-010-1253-9

For peer review only

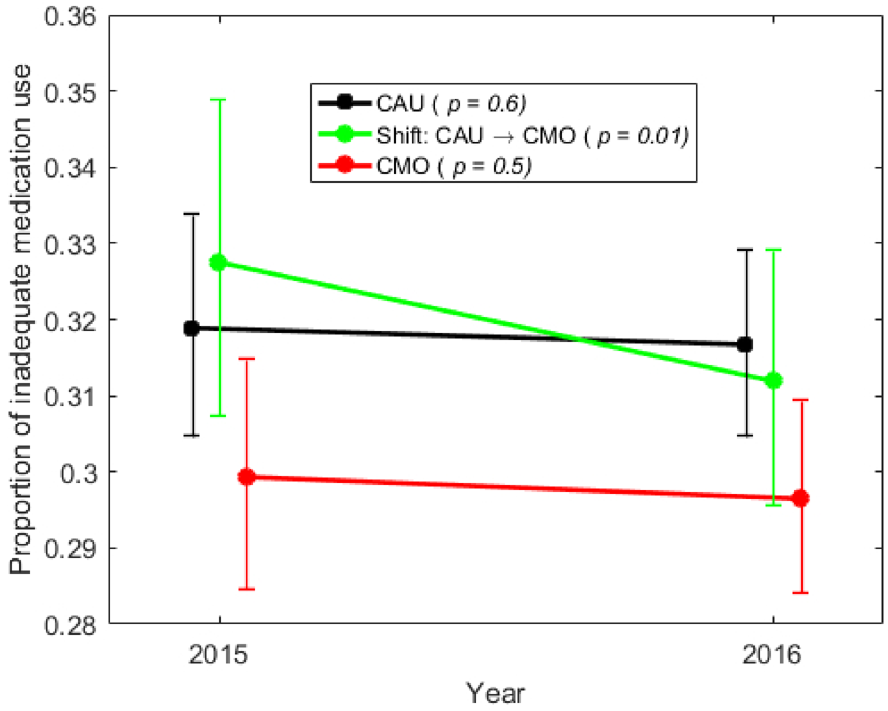

Figure 1. Geometrical mean values (95% CI) for the proportion of inadequate medication use by category of CMO status and year. The p-values indicate the significance of longitudinal changes within each category of CMO status.

BMJ Open

Implementation of a care manager organisation and its association with antidepressant medication patterns – a register-based study of primary care centres in Sweden

Journal:	*BMJ Open*
Manuscript ID	bmjopen-2020-044959.R1
Article Type:	Original research
Date Submitted by the Author:	28-Jan-2021
Complete List of Authors:	Sandheimer, Christine; University of Gothenburg Sahlgrenska Academy, Social medicine, School of Public Health and Community Medicine<br>Björkelund, Cecilia; University of Gothenburg Sahlgrenska Academy, Primary Healthcare, School of Public Health and Community Medicine; Research and Development, Primary Healthcare, Region Västra Götaland<br>Hensing, Gunnel; University of Gothenburg Sahlgrenska Academy, Social medicine, School of Public Health and Community Medicine<br>Mehlig, Kirsten; University of Gothenburg Sahlgrenska Academy, Lifecourse Epidemiology, School of Public Health and Community Medicine, Institute of Medicine<br>Hedenrud, Tove; University of Gothenburg Sahlgrenska Academy, Medicine use & Pharmaceutical policy, School of Public Health and Community Medicine, Institute of Medicine
**Primary Subject Heading**:	General practice / Family practice
Secondary Subject Heading:	Health services research, Mental health, Patient-centred medicine, Public health, Pharmacology and therapeutics
Keywords:	PRIMARY CARE, Quality in health care < HEALTH SERVICES ADMINISTRATION & MANAGEMENT, CLINICAL PHARMACOLOGY, MENTAL HEALTH, Depression & mood disorders < PSYCHIATRY, Anxiety disorders < PSYCHIATRY

SCHOLARONE™
Manuscripts

I, the Submitting Author has the right to grant and does grant on behalf of all authors of the Work (as defined in the below author licence), an exclusive licence and/or a non-exclusive licence for contributions from authors who are: i) UK Crown employees; ii) where BMJ has agreed a CC-BY licence shall apply, and/or iii) in accordance with the terms applicable for US Federal Government officers or employees acting as part of their official duties; on a worldwide, perpetual, irrevocable, royalty-free basis to BMJ Publishing Group Ltd ("BMJ") its licensees and where the relevant Journal is co-owned by BMJ to the co-owners of the Journal, to publish the Work in this journal and any other BMJ products and to exploit all rights, as set out in our licence.

The Submitting Author accepts and understands that any supply made under these terms is made by BMJ to the Submitting Author unless you are acting as an employee on behalf of your employer or a postgraduate student of an affiliated institution which is paying any applicable article publishing charge ("APC") for Open Access articles. Where the Submitting Author wishes to make the Work available on an Open Access basis (and intends to pay the relevant APC), the terms of reuse of such Open Access shall be governed by a Creative Commons licence – details of these licences and which Creative Commons licence will apply to this Work are set out in our licence referred to above.

Other than as permitted in any relevant BMJ Author's Self Archiving Policies, I confirm this Work has not been accepted for publication elsewhere, is not being considered for publication elsewhere and does not duplicate material already published. I confirm all authors consent to publication of this Work and authorise the granting of this licence.

For peer review only

Implementation of a care manager organisation and its association with antidepressant medication patterns – a register-based study of primary care centres in Sweden

Christine Sandheimer[1*], Cecilia Björkelund[2,5], Gunnel Hensing[1], Kirsten Mehlig[3], Tove Hedenrud[4]

Affiliations

[*]Correspondence: Christine.sandheimer@gu.se

[1]Social medicine, School of Public Health and Community Medicine, Institute of Medicine, The Sahlgenska Academy, University of Gothenburg, Gothenburg, Sweden

[2]Primary Healthcare, School of Public Health and Community Medicine, Institute of Medicine, The Sahlgenska Academy, University of Gothenburg, Gothenburg, Sweden

[3]Lifecourse Epidemiology, School of Public Health and Community Medicine, Institute of Medicine, The Sahlgenska Academy, University of Gothenburg, Gothenburg, Sweden

[4]Medicine use & Pharmaceutical policy, School of Public Health and Community Medicine, Institute of Medicine, The Sahlgenska Academy, University of Gothenburg, Gothenburg, Sweden

[5]Research and Development, Primary Healthcare, Region Västra Götaland, Sweden

Word count: 3850

For peer review only

Abstract

Objective To evaluate the implementation of a care manager organisation for common mental disorders and its association with antidepressant medication patterns on primary care centre (PCC) level, compared to PCCs without this organisation. Moreover, to determine whether a care manager organisation is associated with antidepressant medication patterns that is more in accordance with treatment guidelines.

Design Register-based study on PCC level.

Setting Primary care in Region Västra Götaland, Sweden.

Participants All PCCs in the region. PCCs were analysed in three subgroups: PCCs with a care manager organisation during 2015 and 2016 (n=68), PCCs without the organisation (n=92) and PCCs that shifted to a care manager organisation during 2016 (n=42).

Outcome measures Proportion of inadequate medication users, defined as number of patients ≥18 years with a CMD diagnosis receiveing care at a PCC in the region during the study period and were dispensed 1-179 Defined Daily Doses of antidepressants (of total patients with at least 1 DDD). The outcome was analysed through generalised linear regression and a linear mixed-effects model.

Results In both years, all PCCs had about 30 – 34 % of inadequate medication users. PCCs with a care manager organisation had significantly lower proportion of inadequate medication users in 2016 compared to PCCs without (-6.4%, p = .02). These differences were explained by higher proportions in privately run PCCs. PCCs that shifted to a care manager organisation had a significant decrease in inadequate medication users over time (p = .01).

Conclusions Public PCCs had a more consistent antidepressant medication pattern compared to private PCCs that gained more by introducing a care manager organisation. It was possible to evaluate a significant decrease in inadequate medication users, not-withstanding that PCCs

For peer review only - http://bmjopen.bmj.com/site/about/guidelines.xhtml

in the region followed the guidelines to a comparatively high extent regardless of present care manager organisation.

Keywords

Care manager, collaborative care, common mental disorders 'CMD', primary care, care as usual 'CAU', antidepressant medications

Strengths and limitations of this study

- This is the first study aimed at assessing the implementation of a care manager organisation in Swedish ordinary primary care (PC) on a regional level
- Register data on dispensed antidepressant medication per primary care centre (PCC) is a strong quality indicator in the assessment of the PCC's adherence to clinical guidelines
- The use of register data made it possible to include all PCCs in the south-west of Sweden
- As primary healthcare is context bound, the generalisability of the findings from this study may be limited
- As the differences between the PCCs and over time were small, future studies should include a longer time period to be able to distinguish the long-term effects of a care manager organisation in Swedish PC

For peer review only

Introduction

Common mental disorders (CMDs) such as depression, anxiety syndromes, and adjustment and stress-related disorders, are major causes of the disease burden world-wide [1]. In high income countries, such as most European countries and the United States, approximately 18 percent of the population experience a CMD during a year [2]. The economic burden of CMDs contain both direct costs, in the forms of costs of healthcare visits and medications, and indirect costs, such as sickness absence and production loss [3].

When it comes to healthcare visits, individuals with CMDs often seek care at the primary care (PC) as the first contact with the healthcare system and it is in the PC the majority are treated [4–6]. In spite of this, for several years research has shown that the PC systematically fails to identify patients with CMDs [4], especially when the patient seek care for a somatic complaint [7]. Moreover, the treatments for CMDs, the most common being psychotherapy and antidepressant medication use, have been shown to be inadequate and to not always follow established clinical treatment guidelines [8]. A suboptimal use of antidepressant medication increases the risk of relapse and might adversely affect the treatment outcome [9]. Research has also shown that just starting patients on antidepressant medications without the support and continuity given in coordinated and collaborative care only helped a minority of patients with depression [10].

During the last two decades, several countries have started to implement collaborative care models in their PC to improve the care given to patients with CMDs [11,12]. The collaborative care model is an organisational intervention often including four key components: i) an interdisciplinary approach, ii) structured care planning, iii) a systematic follow-up of the patient, and iv) increased communication between healthcare personnel [13]. The model was adapted from the Chronic Care Model of disease management developed by Wagner in 1996 [14]. Wagner and colleagues suggested that team-based care, incorporating

For peer review only - http://bmjopen.bmj.com/site/about/guidelines.xhtml

the four above-mentioned components and where a non-physician caregiver (e.g. a nurse) had the main responsibility for the patient, would make the care more efficient and would improve treatment outcomes [14].

In 2012, the Swedish Council on Health Technology Assessment [15] stressed a need for studies in Swedish PC to determine whether the collaborative care model with special trained care managers could result in the same positive effects as shown in other PC settings [16,17]. Thus, a first pragmatic, cluster-randomised controlled trial (PRIM-CARE) with 23 primary care centres (PCC) in Region Västra Götaland in the south-west of Sweden was conducted in 2014 [18]. The results from the PRIM-CARE study showed positive effects of the care manager organisation regarding symptom remission and a more consistent antidepressant medication use compared to care as usual (CAU) [18]. After the intervention study a new phase was initiated with the aim of implementing a care manager organisation more widely in the region's PCCs.

Even though the positive effects on depression and anxiety outcomes of collaborative care in the PC have been well established by now, the evidence base regarding quality assessment of important components in the collaborative care model is poor [19]. One important process variable for a quality assessment is to measure the continuation of antidepressant medication use [20]. A common variable to measure continuity of medication is Defined Daily Doses (DDD) defined as: *"The assumed averaged maintenance dose per day for a drug used for its main indication in adults."*[21]. According to clinical guidelines, antidepressant medication use for depression and anxiety disorders should continue for at least six months (180 days) to minimise the risk of relapse of symptoms and unnecessary prolongation of the disorder [22,23].

The present study is the first in Sweden that aimed to quality assess the care manager organisation in Swedish PC on PCC level by investigating the association between the

For peer review only

implementation with antidepressant medication patterns. Our objectives were: (1) to investigate antidepressant medication patterns in PCCs with and without a care manager organisation in 2015 (before the implementation) and in 2016 (when the implementation was accomplished), (2) to investigate longitudinal changes in antidepressant medication patterns among the PCCs from 2015 to 2016, and (3) to determine whether establishing a care manager organisation is associated with antidepressant medication patterns more in accordance with established national treatment guidelines.

Methods

Study design and context

The present study was based on aggregated register data at PCC level and was a collaboration between the research programme "New Ways – mental health at work" and the research project PRIM-CARE that is part of the research platform "Ways-of-life, stress and mental health in the primary care context" at the School of Public Health and Community Medicine, University of Gothenburg, Sweden and Research and Development Primary Healthcare, Region Västra Götaland.

Study setting and participating PCCs

This study was conducted in the south-western part of Sweden, in the Region Västra Götaland with a population of 1.7 million (17 % of the Swedish population).

Region Västra Götaland is not just a geographical area in Sweden but also an organisational entity, responsible for financing and producing the healthcare in the region. For the costs of outpatient medications, the region receives an earmarked budget from the state. To ease the economic burden on the individual, the state introduced a cost-containment scheme with a rising scale of state subsidies (10%, 25%, 50%, and 100%), and with a cost-ceiling of 2350

For peer review only

SEK (approx. EUR 220). The cost-ceiling protects the individual from further expenses of outpatient medications during a 12 months period.

The number of PCCs in the region has varied over the years but is around 200, with a small majority having public management. In this study, we included PCCs with an ongoing organisation during both 2015 and 2016 in order to have comparable groups in the statistical analyses.

The care manager organisation

The care manager organisation involves the whole staff at the PCC. All personnel are given brief training on the treatment guidelines for CMDs and how to collaborate with the care manager nurse. The care manager organisation aims at improving the collaboration among personnel, and at providing enhanced patient information and patient education and support about treatment and treatment options. The care manager is a designated nurse that has completed special training in care management for patients with CMDs, especially depression. The work process of the care manager is as follows: when the patient is diagnosed with CMD by the primary care physician, a contact with the care manager nurse is established where a person-centred care plan is drawn up together with the patient. The care plan is followed-up by regular telephone contacts. During the weekly contacts with the patient, that continue for around three months, the care manager assesses the patient's CMD symptoms, and supports and engages the patients in following their treatment, in taking their medications according to the prescription, supervising potential side effects of medications and deciding if there is a need to change the dosage or type of medicine. The care manager have close contact with the treating physician and involves other professionals, like psychotherapists, if needed. The implementation process in Region Västra Götaland started in the fall of 2015, with the first nurses finishing their examination in January 2016. More details about the care manager

For peer review only

function and organisation can be found in a previously published article by Björkelund et al [18].

Care as usual

Care as usual (CAU) for patients with CMDs, in Swedish PCCs, can consist of visits to different healthcare professionals (such as physicians, nurses, psychologists/psychotherapists, or physiotherapists). The treatment should follow the guidelines set by the region which are based on the national evidence-based clinical guidelines for CMDs [24]. These guidelines include face-to-face psychotherapy, antidepressant medication treatment and/or sick listing.

Data and study period

We collected register data on healthcare use and dispensed medications among patients aged 18 and older diagnosed with F32, F33, F40, F41 or F43, (i.e., depression, anxiety syndrome, social phobia and/or stress induced mental ill health) who were receiving care at a PCC in the region within the study period. The data was collected on an aggregated level with information per PCCs and not per patient. The data was obtained from the healthcare database VEGA and the regional prescribed drug register Digitalis. Both are managed by Region Västra Götaland. VEGA contains healthcare information about the inhabitants in the region; all health care consumption is included even the care that has been consumed outside the region. Digitalis contains both information on dispensed medications prescribed in the region from one of the region's PCCs but dispensed in any Swedish pharmacy, as well as information about prescriptions dispensed by the region's inhabitants, even those that are prescribed outside the region.

The study period was January to December 2015 (before implementation of a care manager organisation) and January to December 2016 (when the implementation was accomplished).

For peer review only - http://bmjopen.bmj.com/site/about/guidelines.xhtml

Outcome measure

The outcome measure for this study was number of patients aged 18 and older with a CMD diagnosis who were dispensed at least one antidepressant medication during the study period, as obtained from each PCC. Patients who where dispensed between 1-179 DDD of antidepressant medication (i.e., less than six months treatment) were defined as inadequate medication users in accordance with clinical treatment guidelines for CMDs [22,23]. As outcome measure, we used the *Proportion of inadequate medication users* defined as number of patients with CMD who where dispensed between 1-179 DDD divided by the number of all CMD patients who where dispensed at least 1 DDD. Antidepressants (Anatomical Therapeutic Chemical 'ATC' code N06A) was the medication group of interest in this study as these medications are used in the treatment of both depression and anxiety syndromes [25].

Analytic sample and covariates

The included PCCs were divided into three groups based on their care manager status: (1) PCCs that finished the implementation process of a care manager organisation in January 2016 and continued having the organisation during all of 2016, (2) PCCs that shifted from not having a care manager organisation in 2015 to implementing the organisation from June 2016 and forward, and (3) PCCs without a care manager organisation during the study period (CAU). The following variables were included as potential confounders: i) private or public management; ii) number of listed patients; and iii) proportion of patients with a CMD diagnosis (number of patients with a CMD diagnosis divided by number of listed patients).

Statistical analyses

For peer review only - http://bmjopen.bmj.com/site/about/guidelines.xhtml

The outcome variable *proportion of inadequate medication users* had a distribution that was skewed to the right, and a log-transformation of the variable yielded normally distributed residuals in linear regression models.

Group differences by care manager status were assessed with the non-parametric Kruskal Wallis test and presented with p-values.

Cross-sectional analyses used linear regression of log *proportion of inadequate medication users* on the categories of care manager status (ref = CAU), and further adjusted for private management (ref = public management). The resulting beta-coefficients β were further transformed to give the relative difference in percent for *proportion of inadequate medication users* in PCCs with a care manager organisation compared to PCCs without, calculated as (exp(β) – 1) x 100%. Cross-sectional analyses were performed for both 2015 and 2016.

To investigate the effect of care manager status on the longitudinal change in *proportion of inadequate medication users* within PCC groups we implemented a linear mixed-effects model. The model took into account the correlation between repeated measures for the same PCC [26]. The model for longitudinal change included care manager status, year, the interaction between care manager status and year, and further adjusted for private management status, number of listed patients, and proportion of patients with CMD diagnosis.

Analyses were performed using IBM SPSS Statistics® version 25 and 26, SAS (version 9.4; SAS Institute, Cary, NC) and MATLAB (R2016b; The MathWorks, Inc.). Statistical significance was set at 0.05 (two-sided tests) with presentation of 95 % confidence intervals (CI).

Patient and Public Involvement

Neither patients nor the public were involved in the design or conception of this study.

For peer review only - http://bmjopen.bmj.com/site/about/guidelines.xhtml

Results

Table 1 presents the description of PCCs by care manager status from baseline (2015) and after accomplished implementation (2016). A total of 190 PCCs were included in the study, of which 81 were classified as not having a care manager organisation (CAU), 42 as PCCs that shifted, and 67 as having a care manager organisation. Public or private management (PCC status) was highly correlated with care manager status (*p* = <0.001). The proportions of patients with a CMD diagnosis (of total number of listed patients) per PCC were about 9 % (2015) to 10 % (2016). Visits to nurses and physicians by the study population were higher in 2016 compared to 2015 in all three PCC groups, but only visits to nurses had a statistically significant increase over time across the three categories of care manager status (*p* = <0.01, data not shown). Overall, about 30 to 34% of patients, in all three PCC groups, had less than 180 DDD of antidepressant medication in both 2015 and 2016. PCCs with a care manager organisation had the lowest proportion of inadequate medication users in both 2015 and 2016 compared to the other two PCC groups. All PCC groups had lower proportions of inadequate medication users in 2016 compared to 2015.

Table 1. Description of PCCs by care manager status (CAU[a] n = 81, Shift[b] n = 42 or CMO[c] n = 67) the year before implementation (2015) and the year when the implementation was accomplished (2016).

	**Before implementation (2015)**				**Accomplished implementation (2016)**			
	CAU[a] n (%)	Shift[b] n (%)	CMO[c] n (%)	*p*	CAU n (%)	Shift n (%)	CMO n (%)	*p*
PCC status								
Public	37 (46)	16 (38)	51 (76)	*<.001*	37 (46)	16 (38)	51 (76)	*<.001*
Private	44 (54)	26 (62)	16 (24)		44 (54)	26 (62)	16 (24)	
PCCs with no. of listed patients								
<5000	18 (22)	7 (17)	13 (20)		18 (22)	7 (17)	12 (18)	
5000 - <7000	14 (18)	9 (21)	7 (10)		12 (15)	8 (19)	7 (10)	
7000 - <10.000	26 (32)	17 (41)	21 (31)		26 (32)	18 (43)	23 (35)	
≥ 10.000	23 (28)	9 (21)	26 (39)		25 (31)	9 (21)	25 (37)	
Mean value (SD)	8206 (3631)	8268 (3786)	8855 (3842)	*.53*	8452 (3594)	8442 (3749)	8921 (3802)	*.69*
PCCs with no. of patients with CMD diagnosis								
<500	23 (28)	10 (24)	15 (22)		16 (19)	10 (24)	13 (20)	
500 - <700	18 (23)	13 (31)	14 (21)		20 (25)	6 (14)	9 (13)	
700 - <1000	17 (21)	10 (24)	20 (30)		20 (25)	14 (33)	20 (30)	
≥ 1000	23 (28)	9 (21)	18 (27)		25 (31)	12 (29)	25 (37)	

For peer review only

Mean value (SD)	756 (344)	774 (411)	818 (394)	*.95*	820 (366)	858 (429)	901 (429)	*.25*
PCCs with no. of patients on antidepressants							67	
<300	23 (28)	11 (26)	13 (20)		17 (21)	11 (26)	11 (17)	
300 - <500	24 (30)	18 (43)	23 (34)		30 (37)	14 (34)	21 (31)	
500 - <700	21 (26)	6 (14)	20 (30)		18 (22)	9 (21)	18 (27)	
≥ 700	13 (16)	7 (17)	11 (16)		16 (20)	8 (19)	17 (25)	
Mean value (SD)	450 (223)	461 (241)	485 (233)	*.70*	487 (231)	499 (249)	528 (244)	*.57*
PCCs with no. of visits by patients with CMD to:								
Physicians[e], n[d]	80		66					
<1000	19 (24)	12 (29)	14 (21)		17 (21)	9 (21)	9 (13)	
1000 - <1500	22 (27)	12 (29)	15 (23)		14 (17)	11 (26)	15 (23)	
1500 - <2000	15 (19)	8 (19)	13 (20)		17 (21)	5 (12)	11 (16)	
≥ 2000	24 (30)	10 (23)	24 (36)		33 (41)	17 (41)	32 (48)	
Mean value (SD)	1660 (855)	1584 (994)	1839 (954)	*.27*	1873 (946)	1985 (1245)	2202 (1218)	*.35*
Nurses[e], n[d]	76	36	64		77	37	67	
<50	34 (45)	15 (42)	23 (36)		18 (23)	9 (25)	11 (17)	
50 - <300	22 (29)	15 (42)	17 (26)		25 (33)	19 (51)	25 (37)	
300 - <500	6 (8)	3 (8)	12 (19)		8 (10)	2 (5)	6 (9)	
≥ 500	14 (18)	3 (8)	12 (19)		26 (34)	7 (19)	25 (37)	
Mean value (SD)	293 (453)	191 (322)	291 (450)	*.61*	455 (588)	282 (368)	571 (690)	*.05*
Proportion of inadequate medication users								
% (SD)	32.8 (9.7)	33.8 (10.4)	30.3 (5.1)	*.15*	32.5 (8.3)	31.6 (5.3)	29.9 (4.0)	*.18*

[a]Care as usual, [b]Shifted from CAU to a care manager organisation, [c]Care manager organisation
[d]Number of valid observations

Table 2. Association between the proportion of inadequate medication users and PCCs' care manager status (CAU[a], Shift[b] or CMO[c]).

		**2015** (Baseline) Relative difference[+] % (95% CI)		**2016** Relative difference[+] % (95% CI)	
	n[d]	Unadjusted model	Adjusted for private PCC	Unadjusted model	Adjusted for private PCC
CAU	81	Ref.	Ref.	Ref.	Ref.
Shift	42	2.7% (-5; 11)	1.6% (-5.6; 9.4)	-1.5% (-7.8; 2.5)	-2.5% (-8.3; 3.7)
CMO	67	-6.1% (-12.3; 0.4)	-2% (-8.3; 4.7)	-6.4% (-11.6; -0.9)*	-2.6% (-7.9; 2.9)

[a]Care as usual, [b]Shifted from CAU to a care manager organisation, [c]Care manager organisation
[+]Relative difference in percent = [exp(β)-1] x 100 %.
[d]Number of valid observations
*p-value <0.05

Cross-sectional results

In both years, cross-sectional analyses showed that PCCs with a care manager organisation had about 6 % lower proportion of inadequate medication users compared to PCCs without, but the difference was only significant in 2016 ($p = 0.02$) (Table 2).

For peer review only - http://bmjopen.bmj.com/site/about/guidelines.xhtml

After controlling for private or public management the differences by care manager status reduced. The covariates *number of listed patients* and *proportion of patients with CMD diagnosis* did not have any effect on the association between *proportion of inadequate medication users* and care manager status (data not shown). The association between care manager status and *proportion of inadequate medication users* did not differ between private or public PCCs (*p*-value for interaction > 0.4 for both time points).

Figure 1 here

Longitudinal results

We used a linear mixed-effects model to investigate changes in *proportion of inadequate medication users* between the three PCC groups with different care manager status over time (Fig. 1). The results showed no overall interaction between year and care manager status (p = 0.17, data not shown). As illustrated in figure 1, all three PCC groups had a decrease in *proportion of inadequate medication users* over time but this change was only significant in PCCs that shifted from CAU to a care manager organisation ($p = 0.01$). This trend was also observed after further adjustment for private management ($p = 0.01$) and for the proportion of patients with a CMD diagnosis ($p = 0.02$).

Discussion

The present study is the first in Sweden that, on PCC level, has assessed a new care function with an organisational change in the Swedish primary care, that is, the care manager organisation.

The main finding of this study was that a significantly lower proportion of patients with inadequate antidepressant medication could be shown for the PCCs that had implemented a

For peer review only

care manager organisation from the start compared to PCCs without. This finding was to a large extent explained by PCCs with private management, which showed the greatest impact of implementing a care manager organisation for patients with CMD. Furthermore, only PCCs that shifted from CAU in 2015 to a care manager organisation at some point during 2016 had a significant decrease of inadequate medication users over time. This PCC group was dominated by privately driven PCCs. Among PCCs with a care manager organisation from the start, the change was barely visible as these PCCs had a lower proportion of inadequate antidepressant medication users even before the implementation. Another finding was that publicly driven PCCs with and without a care manager organisation did not differ in proportion of inadequate medication users.

The absence of significant differences between the PCC groups, could possibly be partially explained by the fact that PCCs in Region Västra Götaland are bound by contract to deliver care according to regional clinical guidelines. The region has set up prescribing objectives for a rational medication use [27]. These objectives are found in a prescribing guidelines booklet available for all PCCs to help them develop good prescribing routines. This could be interpreted to mean that even a PCC without a care manager organisation can deliver high pharmacological care quality that resonates well with the guidelines. Our assumption is supported by the findings from a study performed in the same region as our in which primary care physicians' attitudes and use of the prescribing guidelines booklet were investigated [28]. The study found that the majority of physicians had positive attitudes towards the booklet and used it frequently. The same study also showed that physicians in private PCCs were more negative towards the booklet and did not adhere to the guidelines to the same extent as the physicians in the public organisations. This result could partly explain our finding that the public PCCs were stable in their medication use after the implementation of a care manager organisation and mostly the private PCCs showed an improvement.

For peer review only

For peer review only - http://bmjopen.bmj.com/site/about/guidelines.xhtml

Approximately 70 % of the patients with a CMD diagnosis, in all three PCC groups, were dispensed an antidepressant medication for six months or longer (180 DDDs or more), which is in accordance with the recommendations in established clinical guidelines [22,23]. Findings from other studies have presented numbers between 55 % (France) to 66 % (Denmark), and 74 % (Sweden) of antidepressant length lasting for more than six months after remission [29–31]. As an international comparison, Sweden has a high proportion of the population taking antidepressant medications [32–34], and Region Västra Götaland stands out as one of the regions in Sweden with highest prevalence of antidepressant users [35].

A point important to acknowledge is that not all individuals need a medication treatment that lasts more than 180 days. An ambition of 100 % medication use in accordance with treatment guidelines is therefore unrealistic. Research has shown that about 15 % of Swedish patients do not collect their medications and the reasons behind are many [36]. The guidelines should be seen as recommendations not disregarding the patients' individual needs [36].

In the preceding intervention study (of the present study), the result showed that a care manager positively affected the antidepressant treatment in patients with depression [18]. The findings from the intervention study were in line with previous research from other healthcare contexts which has proved that support from a care manager during the treatment for CMDs increases the frequency of adequate antidepressant medication use on the individual level [10,15,20,37].

Methodological considerations

The chosen design for this study possesses both strengths and potential limitations. A strength with a register based study is the reduced risk of bias due to low sampling, high attrition levels, recall bias and other methodological fallacies that are more frequent in survey studies [38]. The register databases used in this study; the healthcare database VEGA and the

For peer review only

regional prescription drug register Digitalis, are comprehensive, systematic and updated on a regular basis. Digitalis is based on the same data as the national prescribed drug register in Sweden, enabling a national comparison of data. The use of data from VEGA and Digitalis allowed us to include all PCCs in the region of various sizes and management types, and from different rural and urban settings. There are approximately 1200 PCCs in Sweden, and this makes our sample, and thus our findings, generalisable on a national level. However, the applicability of our results to an international context could be limited since healthcare systems, and particularly, the primary care organisation, differ widely between countries [15]. A limitation with the use of aggregated data is the risk of information bias that is difficult to control for when we lack detailed information on the individual level [39]. The registers contain comprehensive information but they also require skills and experience of researchers to discern what the statistics embody and if the information is managed correctly. In our case, a thorough scrutiny of the data led to the exclusion of 22 PCCs that had extreme values of inadequate medication users because the PCCs did not have a health care activity (i.e., were in operation) during the whole study period. As a result, we believe the reliability of our findings improved substantially.

Another methodological issue is the small observed differences between the PCC groups that could be due to the chosen period of study. The implementation process for the care manager organisation started in January 2016 but the education for the care manager nurses began in late 2015. To have used 2015 as the baseline year and 2016 as the follow-up year could perhaps have led to the absence of greater differences. A longer follow-up period of potential effects of the introduction of the care manager organisation would have been preferable.

The last potential limitation in the present study is the absence of prescription data, i.e. information about what the physicians prescribed. A comparison between dispensed and prescribed medications to discern possible disparities in the data would have made a stronger

For peer review only - http://bmjopen.bmj.com/site/about/guidelines.xhtml

quality indicator. Nevertheless, dispensed medication is a better indicator of medication use than prescription data [40] as it gives us a clearer picture of the active behavior of the patient rather than the prescriber, and because many patients do not fill their prescriptions [36]. Hence, we believe that register data on dispensed medication was the better option for measurement as one of the tasks of the care manager is to help the patients to take their medicines according to the prescription guidelines.

Implications

Antidepressant medication treatment is influenced by several different factors that affect the outcome. These include both medical and patient-related factors such as accessibility, diagnostics and diagnostic process, guidelines, patient knowledge, and adherence [41,42]. Most of these factors can be influenced by the care manager [19] and thus an assessment of quality improvements achieved by a care manager organisation, at the PCC, should be evaluated not only on the individual patient level, but also on the PCC level by using healthcare registers. Assessments of complex interventions aiming at changing routine practice in primary care form an important knowledge base for decision-makers responsible for the provision and financing of healthcare.

As this is the first study that assessed the implementation of a care manager organisation in Swedish PC on PCC level with data when the organisational change was new, future studies are needed to further investigate the effects of the care manager organisation on a long-term basis.

Conclusions

This quality assessment of the care manager organisation for patients with CMDs in Swedish primary care constitutes an important knowledge base in the further implementation process.

For peer review only

The results of this study showed that public PCCs had a more consistent antidepressant medication use pattern compared to PCCs with private management, that gained more in introducing a care manager organisation. Moreover, that PCCs in Region Västra Götaland, in general, followed the guidelines to a comparatively high extent both with and without the introduction of a care manager organisation. However, future studies should include a longer time period to be able to distinguish the long-term effects of a care manager organisation in Swedish PC.

Acknowledgements

The authors would like to thank Nashmil Arai for her excellent work on the data management.

Author Contributions

CB is the principal investigator and the initiator and guarantor of the project. CB, TH and CS participated in the design of the study. CS, CB, GH, KM and TH took part in planning the analyses. CS handled the data and had the main responsibility for conducting the analyses and for the writing of the paper. KM supervised and assisted in the statistical analysis. CB, GH, KM and TH contributed with the draft and revision of the manuscript. All authors contributed to the interpretation of data and read and approved the final version of this manuscript.

Funding

This work was supported by the Swedish Research Council for Health, Working Life and Welfare (FORTE), grant number 2013-2216, and by grants from the Swedish state under the agreement between the Swedish government and the regions, the ALF-agreement (ALFGBG-722441) and Region Västra Götaland. The funding organ had no influence on the design of

For peer review only

the study, data collection, analysis or interpretation of data or writing of the report or the decision to publish the article. All authors were independent from the funder.

Competing interests

None declared.

Patient consent for publication

Not required.

Ethics approval

The collected register data used in this study was on an aggregated level with information per PCC and not per patient. No individuals could be identified and no informed consent was necessary. Ethical approval was received from the regional Ethical Review Board in Gothenburg, Sweden (Dnr: T566-17).

Provenance and peer review

Not commisioned; externally peer reviewed.

Data availability statement

Data are not publicly available due to Swedish law but are available from the authors upon reasonable request.

Open access

This is an open access article distributed in accordance with the Creative Commons Attribution Non Commercial (CC BY-NC 4.0) license, which permits others to distribute,

For peer review only

For peer review only - http://bmjopen.bmj.com/site/about/guidelines.xhtml

remix, adapt, build upon this work non-commercially, and license their derivative works on different terms, provided the original work is properly cited, appropriate credit is given, any changes made indicated, and the use is non-commercial. See: http://creativecommons.org/licenses/by- nc/4. 0/.

ORCID iDs

Christine Sandheimer http://orcid.org/0000-0002-2429-0669

Cecilia Björkelund http://orcid.org/0000-0003-4083-7342

Gunnel Hensing https://orcid.org/0000-0002-3457-2993

Kirsten Mehlig https://orcid.org/0000-0002-2653-0734

Tove Hedenrud https://orcid.org/0000-0003-2526-7097

Figure caption

Figure 1. Unadjusted model of geometrical mean values (95% CI) for proportion of inadequate medication users by category of care manager status (CAU, Shift, CMO) and year. The p-values indicate the significance of longitudinal changes within each category of care manager status.

References

1 WHO. Depression and Other Common Mental Disorders: Global Health Estimates. *World Heal Organ* 2017.

2 Steel Z, Marnane C, Iranpour C, *et al.* The global prevalence of common mental disorders: A systematic review and meta-analysis 1980-2013. *Int J Epidemiol* 2014;**43**:476–93. doi:10.1093/ije/dyu038

3 OECD. Health at a Glance: Europe 2018 (Summary in Spanish). In: *OECD Publishing*.

2018. doi:10.1787/fd41e65f-es

4 Sundquist J, Ohlsson H, Sundquist K, *et al.* Common adult psychiatric disorders in Swedish primary care where most mental health patients are treated. *BMC Psychiatry* 2017;**17**:1–9. doi:10.1186/s12888-017-1381-4

5 Lykkegaard J, Rosendal M, Brask K, *et al.* Prevalence of persons contacting general practice for psychological stress in Denmark. *Scand J Prim Health Care* 2018;**36**:272–80. doi:10.1080/02813432.2018.1499494

6 Kessler RC, Demler O, Frank RG, *et al.* Prevalence and Treatment of Mental Disorders, 1990 to 2003. *N Engl J Med* 2005;**352**:2515–23.

7 Adamsson A, Bernhardsson S. Symptoms that may be stress-related and lead to exhaustion disorder: A retrospective medical chart review in Swedish primary care. *BMC Fam Pract* 2018;**19**:1–9. doi:10.1186/s12875-018-0858-7

8 Sinnema H, Terluin B, Volker D, *et al.* Factors contributing to the recognition of anxiety and depression in general practice. *BMC Fam Pract* 2018;**19**. doi:10.1186/s12875-018-0784-8

9 Donoghue J, Taylor DM. Suboptimal use of antidepressants in the treatment of depression. *CNS Drugs* 2000;**13**:365–83. doi:10.2165/00023210-200013050-00006

10 DeJesus RS, Angstman KB, Cha SS, *et al.* Antidepressant Medication Use Among Patients with Depression: Comparison between Usual Care and Collaborative Care Using Care Managers. *Clin Pract Epidemiol Ment Heal* 2013;**9**:84–7. doi:10.2174/1745017901309010084

11 Overbeck G, Kousgaard MB, Davidsen AS. The work and challenges of care managers in the implementation of collaborative care: A qualitative study. *J Psychiatr Ment Health Nurs* 2018;**25**:167–75. doi:10.1111/jpm.12449

12 Nieuwenhuijsen K, Faber B, Verbeek J, *et al.* Interventions to improve return to work

For peer review only

in depressed people. *Cochrane Database Syst Rev* 2014;**2014**.

13 Gunn J, Diggens J, Hegarty K, *et al.* A systematic review of complex system interventions designed to increase recovery from depression in primary care. *BMC Health Serv Res* 2006;**6**:1–11. doi:10.1186/1472-6963-6-88

14 Wagner EH, Austin BT, Korff M Von, *et al.* Organizing Care for Patients with Chronic Illness. *Milbank Q* 1996;**74**:511–44.https://www.jstor.org/stable/3350391?seq=1#metadata_info_tab_contents

15 Swedish Council on Health Technology Assessment ('SBU' Statens beredning för medicinsk utvärdering). Implementation support for psychiatric evidence in primary care. Report 211. Stockholm: 2012.

16 Rost K, Nutting P, Smith J, *et al.* Improving depression outcomes in community primary care practice: A randomized trial of the QuEST intervention. *J Gen Intern Med* 2001;**16**:143–9.

17 Gilbody S, Whitty P, Grimshaw J, *et al.* Educational and Organizational Interventions to Improve the Management of Depression in Primary Care: A Systematic Review. *JAMA* 2003;**289**:3145–51.

18 Björkelund C, Svenningsson I, Hange D, *et al.* Clinical effectiveness of care managers in collaborative care for patients with depression in Swedish primary health care: A pragmatic cluster randomized controlled trial. *BMC Fam Pract* 2018;**19**:1–10. doi:10.1186/s12875-018-0711-z

19 Moriarty AS, Coventry PA, Hudson JL, *et al.* The role of relapse prevention for depression in collaborative care: A systematic review. *J Affect Disord* 2020;**265**:618–44. doi:10.1016/j.jad.2019.11.105

20 Hudson JL, Bower P, Kontopantelis E, *et al.* Impact of telephone delivered case-management on the effectiveness of collaborative care for depression and anti-

For peer review only

For peer review only - http://bmjopen.bmj.com/site/about/guidelines.xhtml

depressant use: A systematic review and meta-regression. *PLoS One* 2019;**14**:e0217948. doi:10.1371/journal.pone.0217948

21 WHO. Essential medicines and health products: The ATC/DDD Methodology. 2020.https://www.who.int/medicines/regulation/medicines-safety/toolkit_methodology/en/ (accessed 21 Feb 2020).

22 Cleare A, Pariante CM, Young AH, *et al.* Evidence-based guidelines for treating depressive disorders with antidepressants: A revision of the 2008 British Association for Psychopharmacology guidelines. *J Psychopharmacol* 2015;**29**:459–525. doi:10.1177/0269881115581093

23 Swedish Medical Products Agency [Läkemedelsverket]. Pharmaceutical treatment of depression, anxiety syndrome and OCD amongst children and adults [Läkemedelsbehandling av depression , ångest- syndrom och tvångssyndrom hos barn och vuxna]. *Inf from Swedish Med Prod Agency [Information från Läkemedelsverket Swedish]* 2016;**6:2016**.

24 The Swedish National Board of Health and Welfare [Socialstyrelsen]. *National guidelines for care of depression and anxiety disorders [Vård vid depression och ångestsyndrom]*. Stockholm: : Socialstyrelsen 2020.

25 Swedish Medical Products Agency [Läkemedelsverket]. N06A Antidepressants. Pharm. B. [Läkemedelsboken. Swedish]. 2016.https://lakemedelsboken.se/kapitel/lakemedelsanvandning/trafik_riskfyllt_arbete_och_lakemedel.html?search=N06A&id=N06A-Antidepressiva-medel#N06A-Antidepressiva-medel (accessed 15 Jun 2020).

26 West BT. Analyzing longitudinal data with the linear mixed models procedure in SPSS. *Eval Heal Prof* 2009;**32**:207–28. doi:10.1177/0163278709338554

27 Carlzon D, Gustafsson L, Eriksson AL, *et al.* Characteristics of primary health care

For peer review only - http://bmjopen.bmj.com/site/about/guidelines.xhtml

units with focus on drug information from the pharmaceutical industry and adherence to prescribing objectives: A cross-sectional study. *BMC Clin Pharmacol* 2010;**10**:7–11. doi:10.1186/1472-6904-10-4

28 Axelsson MAB, Spetz M, Mellén A, *et al.* Use of and attitudes towards the prescribing guidelines booklet in primary health care doctors. *BMC Clin Pharmacol* 2008;**8**:1–8. doi:10.1186/1472-6904-8-8

29 Hansen DG. Early discontinuation of antidepressants in general practice: association with patient and prescriber characteristics. *Fam Pract* 2004;**21**:623–9. doi:10.1093/fampra/cmh608

30 Bocquier A, Cortaredona S, Verdoux H, *et al.* Social Inequalities in Early Antidepressant Discontinuation. *Psychiatr Serv* 2014;**65**:618–25. doi:10.1176/appi.ps.201300184

31 Sundell KA, Waern M, Petzold M, *et al.* Socio-economic determinants of early discontinuation of anti-depressant treatment in young adults. *Eur J Public Health* 2013;**23**:433–40. doi:10.1093/eurpub/ckr137

32 Forns J, Pottegård A, Reinders T, *et al.* Antidepressant use in Denmark, Germany, Spain, and Sweden between 2009 and 2014: Incidence and comorbidities of antidepressant initiators. *J Affect Disord* 2019;**249**:242–52. doi:10.1016/j.jad.2019.02.010

33 Gomez-Lumbreras A, Ferrer P, Ballarín E, *et al.* Study of antidepressant use in 5 European settings. Could economic, sociodemographic and cultural determinants be related to their use? *J Affect Disord* 2019;**249**:278–85. doi:10.1016/j.jad.2019.01.039

34 OECD. *Health at a Glance 2013: OECD Indicators*. OECD/Korea Policy Centre 2013. doi:10.1787/9789264261433-ko

35 The Swedish National Board of Health and Welfare [Socialstyrelsen]. Statistical

For peer review only

For peer review only - http://bmjopen.bmj.com/site/about/guidelines.xhtml

database for pharmaceuticals [Statistisk databas för läkemedel]. 2021.https://sdb.socialstyrelsen.se/if_lak/val.aspx (accessed 15 Jan 2021).

36 Freccero C, Sundquist K, Sundquist J, *et al.* Primary adherence to antidepressant prescriptions in primary health care: a population-based study in Sweden. *Scand J Prim Health Care* 2016;**34**:83–8. doi:10.3109/02813432.2015.1132884

37 Hudson TJ, Fortney JC, Pyne JM, *et al.* Reduction of patient-reported antidepressant side effects, by type of collaborative care. *Psychiatr Serv* 2015;**66**:272–8. doi:10.1176/appi.ps.201300570

38 Schwartz S. The fallacy of the ecological fallacy: the potential misuse of a concept and the consequences. *Am J Public Health* 1994;**84**:819–24. doi:10.2105/AJPH.84.5.819

39 Morgenstern H. Ecologic Studies in Epidemiology: Concepts, Principles, and Methods. *Annu Rev Public Health* 1995;**16**:61–81. doi:10.1146/annurev.publhealth.16.1.61

40 Wettermark B, Hammar N, MichaelFored C, *et al.* The new Swedish Prescribed Drug Register—Opportunities for pharmacoepidemiological research and experience from the first six months. *Pharmacoepidemiol Drug Saf* 2007;**16**:726–35. doi:10.1002/pds.1294

41 Kini V, Michael Ho P. Interventions to Improve Medication Adherence: A Review. *JAMA - J Am Med Assoc* 2018;**320**:2461–73. doi:10.1001/jama.2018.19271

42 Fischer MA, Stedman MR, Lii J, *et al.* Primary medication non-adherence: Analysis of 195,930 electronic prescriptions. *J Gen Intern Med* 2010;**25**:284–90. doi:10.1007/s11606-010-1253-9

For peer review only

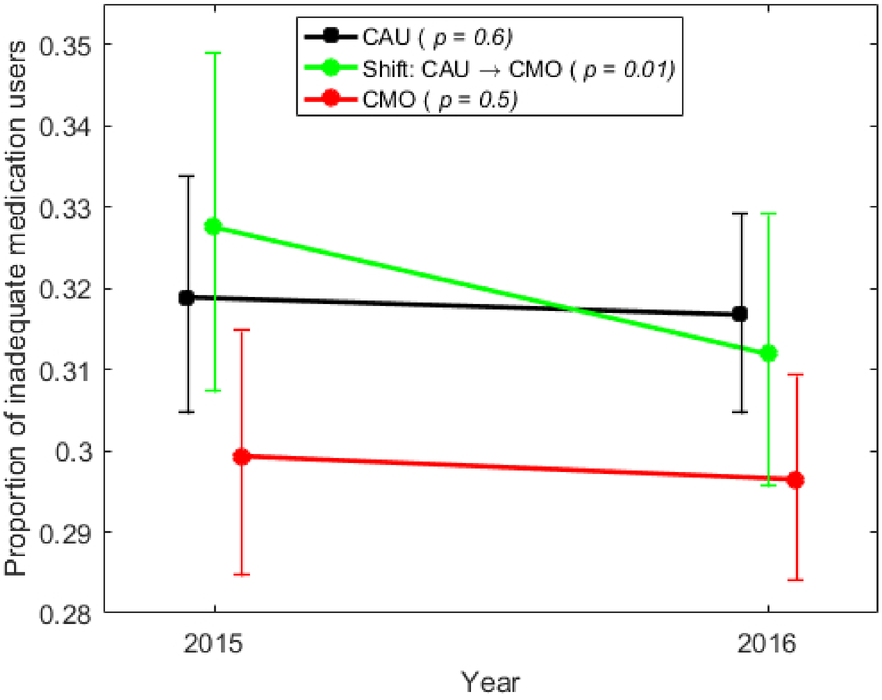

Figure 1. Unadjusted model of geometrical mean values (95% CI) for proportion of inadequate medication users by category of care manager status (CAU, Shift, CMO) and year. The p-values indicate the significance of longitudinal changes within each category of care manager status.

148x111mm (96 x 96 DPI)

STROBE 2007 (v4) checklist of items to be included in reports of observational studies in epidemiology*
Checklist for cohort, case-control, and cross-sectional studies (combined)

Section/Topic	Item #	Recommendation	Reported on page #
**Title and abstract**	1	(*a*) Indicate the study's design with a commonly used term in the title or the abstract	1
		(*b*) Provide in the abstract an informative and balanced summary of what was done and what was found	2
**Introduction**			
Background/rationale	2	Explain the scientific background and rationale for the investigation being reported	4-6
Objectives	3	State specific objectives, including any pre-specified hypotheses	5-6
**Methods**			
Study design	4	Present key elements of study design early in the paper	6
Setting	5	Describe the setting, locations, and relevant dates, including periods of recruitment, exposure, follow-up, and data collection	6-9
Participants	6	(*a*) *Cohort study*—Give the eligibility criteria, and the sources and methods of selection of participants. Describe methods of follow-up<br>*Case-control study*—Give the eligibility criteria, and the sources and methods of case ascertainment and control selection. Give the rationale for the choice of cases and controls<br>*Cross-sectional study*—Give the eligibility criteria, and the sources and methods of selection of participants	7 and 8-9
		(*b*) *Cohort study*—For matched studies, give matching criteria and number of exposed and unexposed<br>*Case-control study*—For matched studies, give matching criteria and the number of controls per case	n/a
Variables	7	Clearly define all outcomes, exposures, predictors, potential confounders, and effect modifiers. Give diagnostic criteria, if applicable	9
Data sources/ measurement	8*	For each variable of interest, give sources of data and details of methods of assessment (measurement). Describe comparability of assessment methods if there is more than one group	8-10
Bias	9	Describe any efforts to address potential sources of bias	n/a
Study size	10	Explain how the study size was arrived at	7
Quantitative variables	11	Explain how quantitative variables were handled in the analyses. If applicable, describe which groupings were chosen and why	10
Statistical methods	12	(*a*) Describe all statistical methods, including those used to control for confounding	9-10
		(*b*) Describe any methods used to examine subgroups and interactions	9-10
		(*c*) Explain how missing data were addressed	n/a
		(*d*) *Cohort study*—If applicable, explain how loss to follow-up was addressed<br>*Case-control study*—If applicable, explain how matching of cases and controls was addressed	n/a

For peer review only

		Cross-sectional study—If applicable, describe analytical methods taking account of sampling strategy	
		(*e*) Describe any sensitivity analyses	n/a
**Results**			
Participants	13*	(a) Report numbers of individuals at each stage of study—eg numbers potentially eligible, examined for eligibility, confirmed eligible, included in the study, completing follow-up, and analysed	11
		(b) Give reasons for non-participation at each stage	n/a
		(c) Consider use of a flow diagram	n/a
Descriptive data	14*	(a) Give characteristics of study participants (eg demographic, clinical, social) and information on exposures and potential confounders	11-12
		(b) Indicate number of participants with missing data for each variable of interest	n/a
		(c) *Cohort study*—Summarise follow-up time (eg, average and total amount)	n/a
Outcome data	15*	*Cohort study*—Report numbers of outcome events or summary measures over time	n/a
		Case-control study—Report numbers in each exposure category, or summary measures of exposure	
		Cross-sectional study—Report numbers of outcome events or summary measures	11-13
Main results	16	(*a*) Give unadjusted estimates and, if applicable, confounder-adjusted estimates and their precision (eg, 95% confidence interval). Make clear which confounders were adjusted for and why they were included	12-13 (Table 2)
		(*b*) Report category boundaries when continuous variables were categorized	n/a
		(*c*) If relevant, consider translating estimates of relative risk into absolute risk for a meaningful time period	n/a
Other analyses	17	Report other analyses done—eg analyses of subgroups and interactions, and sensitivity analyses	13
**Discussion**			
Key results	18	Summarise key results with reference to study objectives	13-15
Limitations	19	Discuss limitations of the study, taking into account sources of potential bias or imprecision. Discuss both direction and magnitude of any potential bias	15-17
Interpretation	20	Give a cautious overall interpretation of results considering objectives, limitations, multiplicity of analyses, results from similar studies, and other relevant evidence	13-15, 17-18
Generalisability	21	Discuss the generalisability (external validity) of the study results	16
**Other information**			
Funding	22	Give the source of funding and the role of the funders for the present study and, if applicable, for the original study on which the present article is based	18

*Give information separately for cases and controls in case-control studies and, if applicable, for exposed and unexposed groups in cohort and cross-sectional studies.

Note: An Explanation and Elaboration article discusses each checklist item and gives methodological background and published examples of transparent reporting. The STROBE checklist is best used in conjunction with this article (freely available on the Web sites of PLoS Medicine at http://www.plosmedicine.org/, Annals of Internal Medicine at http://www.annals.org/, and Epidemiology at http://www.epidem.com/). Information on the STROBE Initiative is available at www.strobe-statement.org.

For peer review only
